# Pharmacokinetics and Safety of Twice-daily Ritonavir-boosted Atazanavir With Rifampicin

**DOI:** 10.1093/cid/ciad700

**Published:** 2023-11-20

**Authors:** Kamunkhwala Gausi, Henry Mugerwa, Marco Siccardi, Maiara Camotti Montanha, Mohammed Lamorde, Lubbe Wiesner, Antonio D’Avolio, Helen McIlleron, Edmund Wilkins, Amedeo De Nicolò, Gary Maartens, Saye Khoo, Cissy Kityo, Paolo Denti, Catriona Waitt

**Affiliations:** Division of Clinical Pharmacology, Department of Medicine, University of Cape Town, Cape Town, South Africa; Joint Clinical Research Centre, Research Department, Kampala, Uganda; Department of Pharmacology and Therapeutics, University of Liverpool, Liverpool, United Kingdom; Department of Pharmacology and Therapeutics, University of Liverpool, Liverpool, United Kingdom; Infectious Diseases Institute, Makerere University College of Health Sciences, Kampala, Uganda; Division of Clinical Pharmacology, Department of Medicine, University of Cape Town, Cape Town, South Africa; Laboratory of Clinical Pharmacology and Pharmacogenetics, Department of Medical Sciences, University of Turin, Turin, Italy; Division of Clinical Pharmacology, Department of Medicine, University of Cape Town, Cape Town, South Africa; North Manchester General Hospital, HIV Research Unit, Manchester, United Kingdom; Laboratory of Clinical Pharmacology and Pharmacogenetics, Department of Medical Sciences, University of Turin, Turin, Italy; Division of Clinical Pharmacology, Department of Medicine, University of Cape Town, Cape Town, South Africa; Department of Pharmacology and Therapeutics, University of Liverpool, Liverpool, United Kingdom; Division of Clinical Pharmacology, Department of Medicine, University of Cape Town, Cape Town, South Africa; Division of Clinical Pharmacology, Department of Medicine, University of Cape Town, Cape Town, South Africa; Department of Pharmacology and Therapeutics, University of Liverpool, Liverpool, United Kingdom

**Keywords:** tuberculosis, HIV, drug-drug interaction, pharmacokinetics, Africa CID specifications

## Abstract

**Background:**

Critical drug-drug interactions (DDI) and hepatotoxicity complicate concurrent use of rifampicin and protease inhibitors. We investigated whether dose escalation of atazanavir/ritonavir could safely overcome the DDI with rifampicin.

**Methods:**

DERIVE (NCT04121195, EDCTP) was a dose-escalation trial in people with human immunodeficiency virus (HIV) on atazanavir/ritonavir-based antiretroviral therapy (ART) in Uganda. Four intensive pharmacokinetic (PK) visits were performed: PK1 300/100 mg OD (baseline); PK2 300/100 mg OD with rifampicin 600 mg; PK3 300/100 mg twice a day (BID) with rifampicin 600 mg OD; PK4 300/100 mg BID with rifampicin 1200 mg OD. Dolutegravir 50 mg BID throughout the study period ensured participants remained protected from subtherapeutic atazanavir concentrations. The data were interpreted with noncompartmental analysis. The target minimum concentration was atazanavir's protein-adjusted IC90 (PA-IC90), 0.014 mg/L.

**Results:**

We enrolled 26 participants (23 female) with median (range) age 44 (28–61) years and weight 67 (50–75) kg. Compared with PK1, atazanavir C_tau_, and AUC were significantly reduced at PK2 by 96% and 85%, respectively. The escalation to BID dosing (PK3) reduced this difference in C_tau,_ and AUC_24_ to 18% lower and 8% higher, respectively. Comparable exposures were maintained with double doses of rifampicin. Lowest C_tau_ during PK1, PK3, and PK4 were 12.7-, 4.8-, and 8.6-fold higher than PA-IC90, respectively, whereas 65% of PK2 C_tau_ were below the limit of quantification (0.03 mg/L), hence likely below PA-IC90. No participant developed significant elevation of liver enzymes, reported a serious adverse event (SAE) or experienced rebound viraemia.

**Conclusions:**

Twice daily atazanavir/ritonavir during rifampicin co-administration was well tolerated and achieved plasma concentrations above the target.

**Clinical Trials Registration:**

NCT04121195. Registered on 09 October 2019, https://clinicaltrials.gov/ct2/show/NCT04121195.

## Teaser

DERIVE, a dose-escalation trial in people with human immunodeficiency virus (HIV) demonstrated that atazanavir/ritonavir (300/100 mg) twice daily could safely overcome the drug-drug interactions (DDI) with rifampicin, as it was well tolerated and achieved plasma concentrations above the target.

World Health Organization (WHO)-recommended treatment options for second-line antiretroviral therapy (ART) in Africa largely consist of a backbone of 2 nucleoside reverse transcriptase inhibitors (NRTIs) combined with ritonavir-boosted protease inhibitors (bPI) [1]. Atazanavir/ritonavir (ATV/r) remains the preferred bPI for many countries, driven by better tolerability compared to lopinavir/ritonavir (LPV/r) [2], and once-daily administration [2]. In 2019, WHO first-line ART changed to dolutegravir, an integrase strand transfer inhibitor (INSTI). However, for individuals who develop resistance [3, 4] or treatment-emergent toxicity on DTG [5], bPIs continue to have a significant role in the management of human immunodeficiency virus (HIV) in sub-Saharan Africa.

Tuberculosis (TB) causes 30% of HIV-related deaths [6] and incidence remains high in people with HIV (PWHIV) despite immune reconstitution on ART [7]. The WHO-recommended first-line TB treatment regimen consists of rifampicin, isoniazid, pyrazinamide, and ethambutol. Recent studies indicate that higher doses of rifampicin lead to more rapid sputum sterilization without increasing toxicity [8]. However, managing TB and HIV concurrently in individuals on second-line ART is challenging due to clinically significant drug-drug interactions (DDI) between rifampicin and bPIs [9] and the high risk of hepatotoxicity when the two are co-administered [10–13].

Rifampicin is a potent inducer of multiple drug-metabolising enzymes, notably cytochrome P450 (CYP) 34A, which metabolizes bPIs, and efflux transporters [14] and the added effect of higher dose on the induction is being explored [15]. The alternative rifamycin, rifabutin is associated with less DDI, but cost, toxicity, and lack of co-formulated preparations render it unlikely as a sustainable option [9].

Previous studies of higher doses of bPI to overcome the DDI effect with rifampicin in healthy volunteers resulted in high rates of hepatotoxicity and eventual premature termination of the trials [16–18]. Recognizing the physiological and pharmacological differences between a drug-naïve healthy volunteer population and PWHIV already established on ART, a 2012 study enrolled PWHIV stable on LPV/r-based regimens [10]. It demonstrated that adjusted doses of LPV/r co-administered with rifampicin-based TB treatment were tolerated. In contrast, a recent attempt to evaluate an increased dose of the bPI, ritonavir-boosted darunavir (DRV/r) combined with rifampicin resulted in rates of hepatotoxicity requiring the trial to be halted [19]. The choice of population (healthy volunteers vs PWHIV vs PWHIV + TB), sequence of introduction of interacting drugs and time for equilibration may have influenced hepatotoxicity risk.

DERIVE (a phase 3, open-label, Dose Escalation study to determine the pharmacokinetics of atazanavir administered with RIfampicin to HIV positive adults on sEcond-line ART regimen with suppressed human immunodeficiency virus type 1 [HIV-1] viral load) study was designed to determine the optimal dose of ATV/r when co-administered with rifampicin-. For accurate dose selection, a Physiologically Based Pharmacokinetic (PBPK) model was built at the University of Liverpool [20]. This model predicted that increasing the dose of ATV/r from 300/100 mg once daily to 300/100 mg twice daily would achieve sufficient plasma concentrations when used together with standard dose rifampicin.

## METHODS

### Study Design

DERIVE (NCT04121195, EDCTP) was an open-label, single-arm, dose-escalation study. Ethical approval to conduct the study was obtained from Joint Clinical Research Centre Research (JCRC) Ethics Committee, the Uganda National Council for Science and Technology, and the Ugandan National Drug Authority. All participants voluntarily gave written informed consent.

### Study Participants

Participants were enrolled from the JCRC in Kampala. Adults with HIV (≥18 and ≤65 years) treated with an ART regimen comprising ATV/r and 2 NRTI for at least 6 months with undetectable viral load (<50 copies/mL) were recruited. All participants had baseline liver enzymes and renal function within normal range. Active tuberculosis was excluded through clinical history, examination, and chest X-ray. Other exclusion criteria were active hepatitis B, concomitant medication with known major interactions with study drugs, or women who were pregnant, breastfeeding, or not on effective contraception.

### Study Procedures

**Figure 1.**
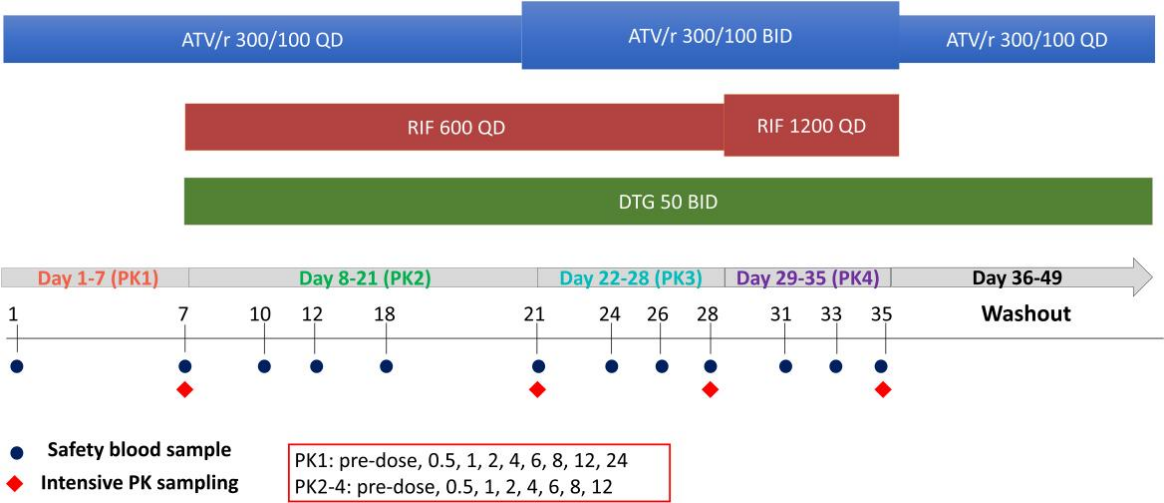
Diagram of the study design. Abbreviations: LLOQ, lower limit of quantification; MEC, minimum effective concentration.

A dose-escalation study design was chosen to evaluate the enzyme-inducing impact of rifampicin on steady-state ATV/r concentrations by comparing them to baseline values. Treatment was open label and distributed at weekly intervals up to day 42 in 5 different steps (Figure 1). Standard doses of NRTIs were maintained throughout the study. A steady-state PK intensive pharmacokinetic profile was performed on day 7 after enrollment (PK1). Rifampicin 600 mg once daily was introduced on day 8, concomitantly with dolutegravir 50 mg twice daily [21], which was added to the regimen for the study duration to prevent risk of the emergence of drug-resistance due to potentially sub-therapeutic atazanavir concentrations. A follow-on PK evaluation was carried out on day 21 (PK2), ATV/r dose was then increased on day 22 in a single step to the PBPK model-predicted dose of 300/100 mg twice a day [20], and another PK evaluation was undertaken on day 28 (PK3). Rifampicin daily dose was then doubled to 1200 mg on day 29 for a further seven days, with further PK done on day 35 (PK4). Thereafter, rifampicin was stopped, ATV/r stepped down to 300/100 mg once a day, while dolutegravir continued for a further 2 weeks up to day 49, although the residual effect of rifampicin induction waned. Participants were followed and assessed for safety measures up to day 60, when they officially exited the study.

### PK Sampling and Assay Procedures

At each PK visits (Days 7, 21, 28, and 35, ie, the last days of each sequential treatment condition; see Figure 1) participants were given the study medications following at least 8 hours of fasting. In total, 4 mL blood samples were drawn just before and 0.5, 1, 2, 4, 6, 8, and 12 hours after the dose. An extra sample 24hour post-dose was drawn in PK1. Blood was centrifuged at 1900*g* to separate plasma, which was stored at −80°C until shipment to Clinical PK Laboratory in the Division of Clinical Pharmacology, University of Cape Town for analysis. Rifampicin assay calibration range was 0.117–30.0 mg/L, with interday accuracy 100.7%–106.6%, and precision coefficient of variation (%CV) 2.7%–13.7% [22]. Plasma concentrations of atazanavir, dolutegravir, and ritonavir were analyzed using a validated multiplex assay, consisting of a liquid-liquid extraction followed by high performance liquid chromatography with tandem mass spectrometry detection. Internal standards were atazanavir-d5, dolutegravir-d4 and ritonavir-d6. An AB Sciex API 4000 mass spectrometer at unit resolution in the multiple reaction monitoring mode was used to monitor the transition of protonated precursor ions. The calibration curves fitted quadratic regressions (weighted by 1/x concentration) over the range 0.030–10.0 mg/L for atazanavir and dolutegravir, and of 0.005 to 2.50 mg/L for ritonavir. Further details are in the Supplementary File.

### Safety Measures

At least 2 laboratory assessments including serum chemistry and full blood count were done between each intensive PK (Days 1, 7, 10, 12, 15, 18, 21, 24, 26, 28, 31, 33, and 35). Individual discontinuation was planned should a participant experience an alanine aminotransferase (ALT) >3× upper limit of normal range and stopping criteria for the trial were defined. An Independent Data Safety Monitoring Board (IDSMB) met after the first 2 participants completed the 35-day dose escalation and again after 5, 15, and 26 participants had completed study procedures.

### Statistical Analysis

From published AUC and SD of atazanavir [23], we predicted that 24 participants would provide 95% power to detect a 20% decrease between the model-predicted dose with rifampicin co-administration and the standard regimen at baseline. R (version 4.1.1) software and ncappc package [24] were used to perform non-compartmental analysis (NCA) and estimate the PK parameters: C_max_, and C_12_ or C_24_ (as appropriate) were observed from the curve, linear up-log down method was used for the calculation of AUC_tau_ and T1/2. In PK2, sampling was up to 12 hours despite 24-hourly dosing for ATV/r, so profiles were extrapolated to 24 hours to calculate AUC_0–24_ and predict C_24_. The same was done for rifampicin in PK2, PK3, and PK4. In PK3 and PK4 (12-hourly dosing), AUC_0–24_ for ATV/r and dolutegravir were calculated by doubling the AUC_0–12_. C_tau_ was determined as the observed C_12_ for PK3 and PK4, C_24_ for PK1, or the predicted C_24_ for PK2. For each drug, concentrations below the lower limit of quantification (LLOQ) of the respective assay were considered as LLOQ/2, except for the calculation of half-life (T_1/2_), for which they were ignored.

Log-transformed PK measures were used in a paired t-test with back-transformation to obtain the point estimate of geometric mean ratio (experimental approaches vs standard doses without rifampicin) and 90% confidence intervals. Proportion of participants with atazanavir C_tau_ below the primary target, which was protein adjusted-IC90 (PA-IC90) of 0.014 mg/L [25], and below the secondary target, which was the minimum effective concentration (MEC) of 0.15 mg/L [26], were described. Atazanavir C_tau_ below the LLOQ were considered below the PA-IC90. Cochran's Q Test was used to assess if there is a difference in the proportion of individuals with C_tau_ below the two targets.

## RESULTS

### Study Population

In total, 26 participants (23 female) were enrolled, and all completed the study, with median (range) age and weight 44 (28–61) years, and 67 (50–75) kg, respectively. Table 1 summarizes baseline characteristics All PK profiles of a single participant were excluded from analysis due to anomalous PK profile during PK1. However, the participant exhibited normal profiles at PK2, PK3, and PK4.s.

**Table 1.** Baseline Characteristics of the Enrolled Cohort (n = 26)

Patient Characteristics	Median Value (Range)
Sex (M/F)	3/23
Age (y)	44 (26–61)
Weight (kg)	67 (50–75)
Baseline ALT (U/L)	14.0 (6.6–33.0)
Baseline AST (U/L)	21.6 (12.4–29.0)
Co-administered NRTIs	
3TC/TDF	17
3TC/AZT	8
3TC/ABC	1

Abbreviations: ALT, alanine aminotransferase; AST, aspartate aminotransferase.

### Pharmacokinetics

**Figure 2.**
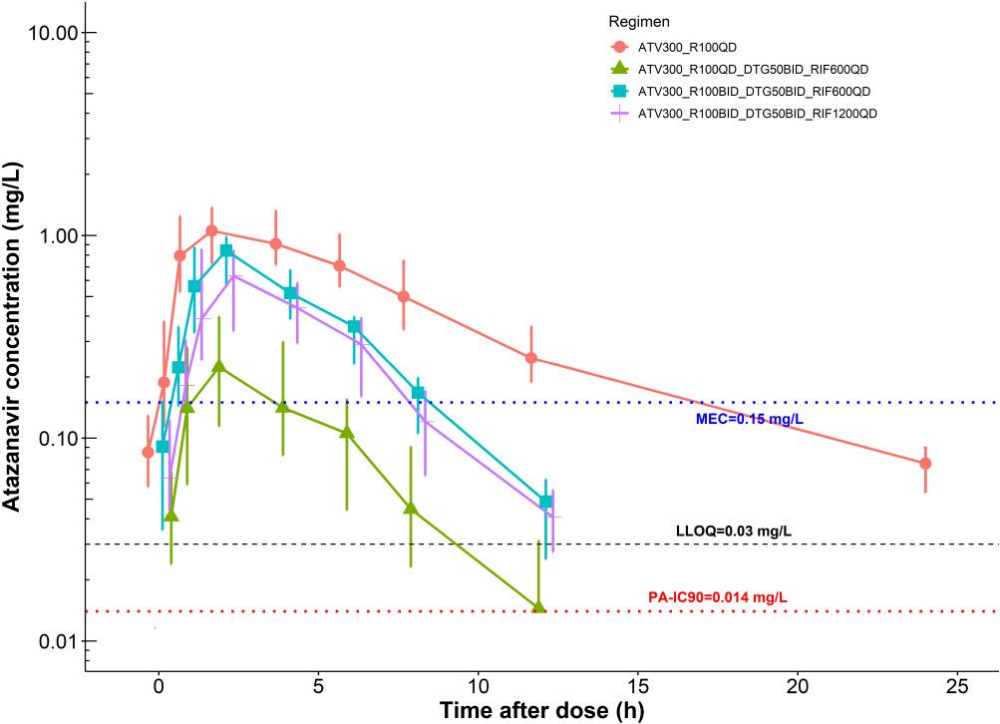
Atazanavir concentration-time profile (median and interquartile range) across the 4 PK visits. The solid lines present observed data, whereas the dashed lines present a repetition of the first 12 h for twice-daily dosing and the extrapolated 24-h concentration for the once-daily dosing profile to ease the visual interpretation of the results. For twice-daily dosing the predose (0 h) and the 12-h concentration are not exactly the same; hence the dashed line does not overlap with the solid one. The two dotted horizontal lines and horizontal dashed lines represent atazanavir minimal effective concentration, protein-adjusted inhibitory concentration 90, and the lower limit of quantification of the assay, respectively.

**Figure 3.**
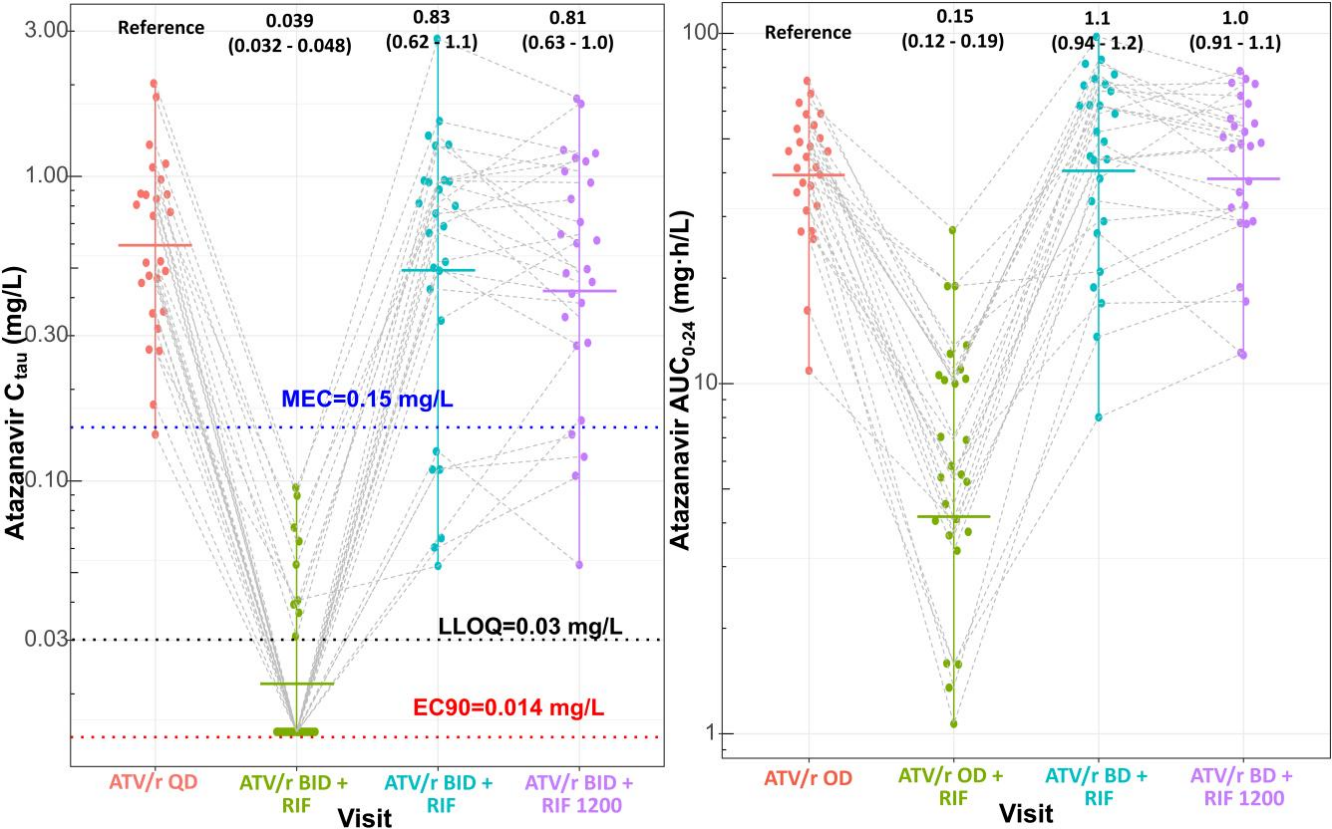
Atazanavir C_tau_ and AUC_0–24_ across the 4 visits. The dots are the individual values of C_tau_ or AUC_0–24_, the horizontal line represents each visit's geometric mean. The values at the top are the geometric mean ratio compared to the baseline visit (PK1). The blue, red, and black dotted lines represent the MEC and EC90 of atazanavir, and the (LLOQ of the assay. Values below LLOQ were imputed as LLOQ/2, which skewed the distribution of C_tau_ in PK2 and affected its geometric mean ratio. Abbreviations: LLOQ, lower limit of quantification; MEC, minimum effective concentration.

Plasma concentration-time profiles of atazanavir across the 4 PK visits are shown in Figure 2, with pharmacokinetic parameters summarized in Table 2. Co-administration of ATV/r 300/100 mg OD with rifampicin 600 mg OD (PK2) significantly reduced atazanavir geometric mean C_tau_, and AUC_24_ by 96% and 85%, respectively. The twice a day (BID) ATV/r dosing (PK3) exposures were not significantly different from those observed during the baseline visit (PK1- without rifampicin co-administration); C_tau_ was 18% lower and AUC_24_ 8% higher. These comparable exposures were maintained with doubling the rifampicin dose to 1200 mg OD (PK4). Figure 3 displays the C_tau_ and AUC_24_ of atazanavir across the 4 PK visits.

**Table 2.** Atazanavir Pharmacokinetic Parameters

PK Parameter	Geometric Mean (90% CI)				Geometric Mean Ratio (90% CI)			
	ATV/r 300/100 QD (PK1)	ATV/r 300/100 QD + RIF 600 (PK2)	ATV/r 300/100 BID + RIF 600 (PK3)	ATV/r 300/100 BID + RIF 1200 (PK4)	PK2 Versus PK1	PK3 Versus PK1	PK4 Versus PK1	PK4 Versus PK3
C_tau_ (mg/L)	0.59 (0.48–0.74)	0.023 (0.015–0.029)	0.49 (0.34–0.70)	0.48 (0.36–0.65)	**0.039 (0.032**– **0.048)**	0.83 (0.64–1.1)	0.81 (0.63–1.0)	0.98 (0.81–1.2)
AUC_24_ (mg·h/L)	40 (35–46)	5.9 (4.5–7.7)	43 (35–53)	40 (33–48)	**0.15 (0.12**–**0.19)**	1.0 (0.94–1.2)	1.0 (0.91–1.1)	0.93 (0.83–1.0)
C_max_ (mg/L)	4.6 (4.0–5.3)	1.2 (0.98–1.5)	3.8 (3.2–4.7)	3.3 (2.8–3.9)	**0.26 (0.20**–**0.33)**	**0.83 (0.70**–**0.99)**	**0.71 (0.64**–**0.79)**	**0.86 (0.75**–**0.98)**
Clearance/F (L/h)	7.5 (6.6–8.7)	50 (39–67)	14 (11–17)	15 (13–18)	**6.8 (5.3**–**8.6)**	**1.8 (1.6**–**2.1)**	**1.9 (1.8**–**2.2)**	1.0 (0.95–1.2)
T_1/2_ (h)	11 (10–12)	2.5 (2.3–2.8)	3.8 (3.4–4.3)	4.1 (3.6–4.6)	**0.22 (0.20**–**0.24)**	**0.34 (0.30**–**0.37)**	**0.36 (0.32**–**0.39)**	1.1 (0.96–1.2)

The bold values represent the significant geometric mean ratio.Abbreviations: ATV/r, atazanavir/ritonavir; AUC, area under the curve; CI, confidence interval.

All participants had concentrations above the PA-IC90 during all PK visits except PK2. The lowest C_tau_ during PK1, PK3, and PK4 were 12.7-, 4.8-, and 8.6-fold higher than PA-IC90, respectively. Values below LLOQ were assumed below PA-IC90. The percentage of C_tau_ below MEC was 4%, 19%, and 8% at PK1, PK3, and PK4. No statistical difference existed in the proportions of individuals with C_tau_ below the MEC target across PK1, PK3, and PK4 (overall *P* value comparing all 3 groups was >.11 and the post-hoc pairwise tests' smallest *P*-value was >.07).

Plasma concentration-time profiles for ritonavir are presented in Supplementary Figure 1 and Supplementary Table 1. Ritonavir exposures (C_tau_ and AUC_24_) decreased by 94% and 87% after introducing rifampicin (PK1 vs PK2). Unlike atazanavir, ritonavir exposure after the dose escalation (PK3) remained significantly lower than the baseline visit (PK1), which persisted with doubling of rifampicin dose (PK4).

**Figure 4.**
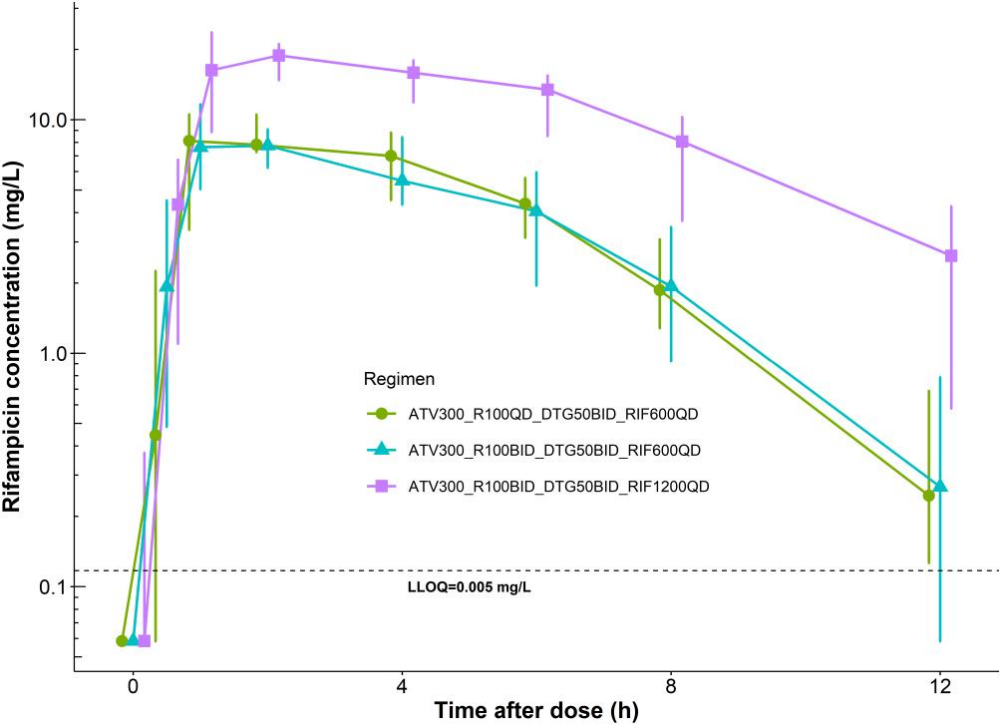
Rifampicin concentration-time profile (median and interquartile range) across the 3 PK visits (PK2–4). The black dashed horizontal line represents the lower limit of quantification (LLOQ) of the assay. Abbreviation: PK, pharmacokinetic.

**Figure 5.**
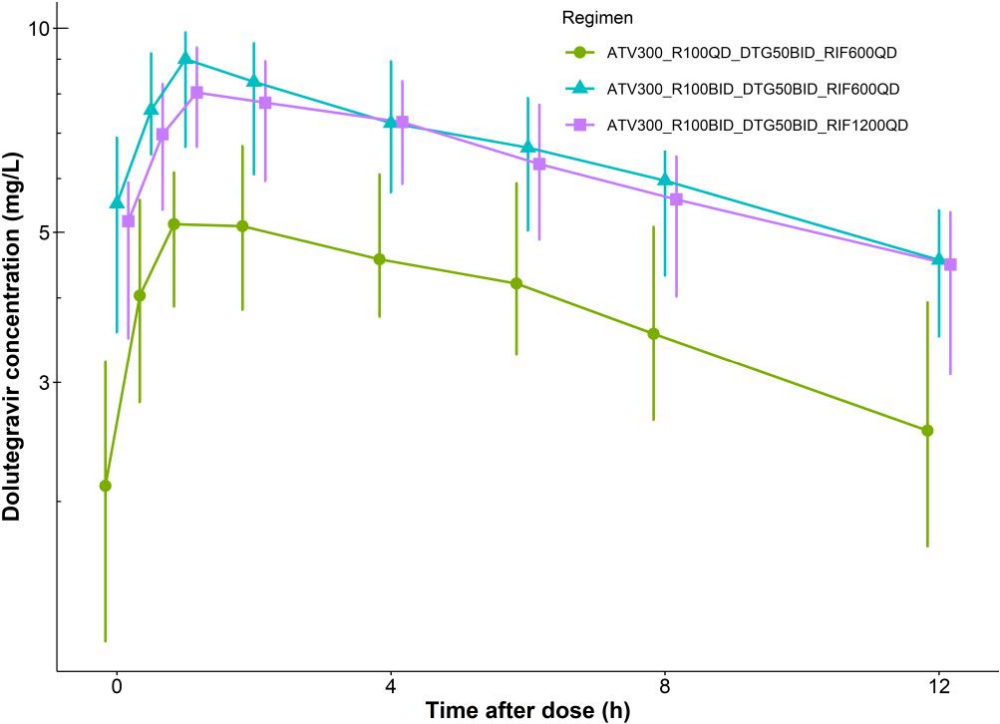
Dolutegravir concentration-time profile (median and interquartile range) across the 3 PK visits (PK2–4). Abbreviation: PK, pharmacokinetic.

The C_tau_ of rifampicin was 40% higher with the dose escalated ATV/r (PK3) compared to the standard dose (PK2), whereas the AUC_24_ and T_1/2_ were similar between the 2 visits. Doubling rifampicin dose resulted in a nonlinear increase in its exposure of 2.7-fold in both C_tau_ and AUC_24_, (PK2 vs PK4). Table 3 summarizes rifampicin pharmacokinetics, and the median plasma concentration-time profiles are shown in Figure 4. Similarly, dolutegravir exposure was higher with the dose escalation as illustrated in Table 4 and Figure 5.

**Table 3.** Rifampicin Pharmacokinetic Parameters

PK Parameter	Geometric Mean (90% CI)			Geometric Mean Ratio (90% CI)		
	ATV/r 300/100 QD + RIF 600 (PK2)	ATV/r 300/100 BID + RIF 600 (PK3)	ATV/r 300/100 BID + RIF 1200 (PK4)	PK3 Versus PK2	PK4 Versus PK2	PK4 Versus PK3
C_12_ (mg/L)	0.057 (0.059–0.059)	0.082 (0.068–0.10)	0.16 (0.11–0.23)	**1.4 (1.2**–**1.7)**	**2.7 (1.85**–**4.0)**	**1.9 (1.5**–**2.5)**
AUC_24_^a^ (mg·h/L)	45 (40–51)	43 (39–48)	117 (102–130)	0.96 (0.89–1.0)	**2.7 (2.3**–**3.0)**	**2.7 (2.5**–**3.0)**
C_max_ (mg/L)	9.7 (8.8–11)	9.5 (88–10)	20 (17–22)	0.98 (0.90–1.1)	**2.0 (1.8**–**2.2)**	**2.0 (1.8**–**2.3)**
Clearance (L/h)	13 (12–15)	15 (14–16)	11 (9.1–12)	1.1 (1.0–1.2)	**0.77 (0.65**–**0.88)**	**0.72 (065**–**0.80)**
T_1/2_ (h)	1.6 (1.5–1.8)	1.6 (1.5–1.8)	2.3 (2.1–2.6)	1.0 (0.98–1.0)	**1.4 (1.3**–**1.5)**	**1.4 (1.3**–**1.5)**

The bold values represent the significant geometric mean ratio.Abbreviations: ATV/r, atazanavir/ritonavir; AUC, area under the curve; BID, twice a day; CI, confidence interval; RIF, rifampicin.

^a^AUC_24_ was derived using extrapolated C_24_.

**Table 4.** Dolutegravir Pharmacokinetic Parameters

PK Parameter	Geometric Mean (90% CI)			Geometric Mean Ratio (90% CI)		
	ATV/r 300/100 QD + RIF 600 (PK2)	ATV/r 300/100 BID + RIF 600 (PK3)	ATV/r 300/100 BID + RIF 1200 (PK4)	PK3 Versus PK2	PK4 Versus PK2	PK4 Versus PK3
C_12_ (mg/L)	2.0 (1.6–2.4)	4.0 (3.4–4.7)	3.8 (3.3–4.5)	**2.0 (1.7**–**2.3)**	1.9 (1.7–2.2)	0.96 (0.85–1.1)
AUC_24_ (mg·h/L)	48 (42–54)	75 (67–84)	71 (64–79)	**1.6 (1.4**–**1.7)**	**1.5 (1.9**–**2.3)**	0.95 (0.89–1.1)
C_max_ (mg/L)	5.5 (4.9–6.2)	8.6 (7.7–9.6)	8.1 (7.3–9.0)	**1.6 (1.4**–**1.7)**	**1.47 (1.34**–**1.60)**	**0.94 (0.88–1.0)**
Clearance (L/h)	1.1 (0.93–1.2)	0.67 (0.93–1.2)	0.71 (0.64–0.79)	**0.64 (0.57**–**0.71)**	**0.68 (0.62**–**0.74)**	1.1 (0.99–1.1)
T_1/2_ (h)	8.7 (7.4–10)	10 (9.3–12)	11 (9.0–1.5)	**1.2 (1.1**–**1.4)**	**1.3 (1.1**–**1.6)**	1.1 (0.84–1.3)

The bold values represent the significant geometric mean ratio.Abbreviations: ATV/r, atazanavir/ritonavir; AUC, area under the curve; BID, twice a day; CI, confidence interval; RIF, rifampicin.

### Safety

No SAEs were observed, and no participant discontinued the study (Table 5). Five participants had transient grade 1 elevations of aspartate aminotransferase (AST) or ALT, which resolved during the study and were asymptomatic: 1 elevated ALT (PK2), 2 had both elevated ALT and 2 AST (PK3), and 2 elevated AST (PK4). No participants developed rebound viraemia.

**Table 5.** Safety Results

Parameter	No. of Subjects (%) for Regimen			
	ATV/r 300/100 q.d (ATV OD)	ATV/r 300/100 q.d + RIF 600 (ATV OD + RIF)	ATV/r 300/100 b.d.q + RIF 600 (ATV BID + RIF)	ATV/r 300/100 b.d.q + RIF 1200 (ATV BID + RIF 1200)
Death	0	0	0	0
Discontinuations due to AEs	0	0	0	0
ALT grade 1 (>1.5 × ULN)	0	1	2	0
AST grade 1 (>1.5 × ULN)	0	0	2	2

Abbreviations: AE, adverse effect; ALT, alanine aminotransferase; AST, aspartate aminotransferase; ATV/r, atazanavir/ritonavir; AUC, area under the curve; BID, twice a day; CI, confidence interval; OD, once daily; RIF, rifampicin.

## DISCUSSION

DERIVE demonstrates that increasing ritonavir-boosted atazanavir to twice-daily dosing, in conjunction with rifampicin, can successfully mitigate the DDI between these 2 drugs. Twice-daily dosing attained trough concentrations that surpass PA-IC90 by ≥4.8 fold and the overall exposure was comparable with that observed at baseline (prior to concomitant rifampicin administration). Furthermore, this approach was well tolerated, particularly regarding absence of hepatotoxic effects. This has clinical relevance, as management of TB in individuals receiving PI-based HIV therapy presents a complex challenge.

Atazanavir is a substrate of both CYP3A4 and p-glycoprotein, which are induced by rifampicin through the activation of nuclear pregnane X receptor [14] and inhibited by ritonavir [27]. Acosta et al [12] predicted unboosted atazanavir (300 mg BID) AUC_24_ and C_12_ geometric mean to decrease by 80% and 95%, respectively, with rifampicin co-administration. Similarly, rifampicin-induced decline in boosted atazanavir (300/100 mg once daily) was estimated to be 72% and 98% by Burger et al [13], which aligns with our findings of 85% and 96% decline in AUC_24_ and C_min_, respectively at PK2. Their escalation of ATV/r dose to 400/200 mg with rifampicin reduced the decline in AUC_24_ to 26%, but C_min_ remained low, with an 89% reduction [13]. They suggested further dose increase, with consideration of twice daily dosing, should be attempted. DERIVE demonstrated that with dose escalation by twice daily administration, both AUC_24_ and C_min_ were not significantly different to the baseline (PK1). Furthermore, our results align with the PBPK prediction of this DDI presented by Montanha and colleagues [20].

Our lowest PK3 C_tau_ exposures remained 4.8-fold greater than PA-IC90, which was our primary target. Increasing rifampicin dose to 1200 mg did not substantially affect exposure, with the lowest C_tau_ during PK4 remaining 8.6-fold higher than PA-IC90. During PK3, 19% of the participants had C_tau_ below the secondary target, MEC of 0.15 mg/L, not significantly different (*P* > .07) from the proportion at PK1 (4%) and PK4 (8%). Importantly, the validity of MEC target has been questioned [28], being based on limited data in participants with multiple PI-related mutations who were not virologically suppressed at initiation. These data are available only as a conference proceeding abstract rather than a substantive peer-reviewed publication [26]. Furthermore, studies [28–30] have shown good virological efficacy in individuals with exposures below the MEC, supporting the use of the more reliable and reproducible PA-IC90 as the primary minimum concentration target.

Severe ALT elevations have been reported when rifampicin was co-administered with bPI including saquinavir, atazanavir, darunavir, and lopinavir [16–19]. However, in DERIVE, the regimen was well tolerated, with no significant transaminase rises or clinical hepatotoxicity. Several factors, as proposed by Decloedt et al [10], may explain this. First, a lower rate of hepatotoxicity has been observed in PWHIV compared with those without HIV when administered rifampicin and pyrazinamide [10, 31–33]. Second, the order of drug introduction appears to be important high rates of hepatotoxicity were observed when rifampicin was introduced prior to bPIs [16–18], hence our choice of participant population and dose escalation strategy. Finally, DERIVE enrolled participants who were stabilized on ATV/r, and elevated ALT was an exclusion criterion.

Dolutegravir was included as a protective measure to mitigate the risk of potential subtherapeutic exposure to atazanavir. This allowed investigation of its PK and DDI with rifampicin and ARV/r. Rifampicin is known to greatly reduce dolutegravir exposure [34], so the latter was dosed twice daily. The AUC of dolutegravir at PK2 was higher than previous reports of dolutegravir BID co-administered with rifampicin, but lower than when given alone (without rifampicin or bPIs [34–38]. Doubling ATV/r frequency resulted in a 1.6-fold increase in dolutegravir exposure. This is predictable: dolutegravir is primarily metabolized through glucuronidation by UGT1A1, with a minor contribution from CYP3A4, and atazanavir is a potent inhibitor of UGT1A1 [39], whereas ritonavir is a potent inhibitor of CYP3A4. A previous report showed atazanavir co-administration led to a 91% increase in dolutegravir exposure [40].

Our observed AUCs for rifampicin during PK2 were within the range and variability previously reported by Daskapan et al [41]. As anticipated, doubling rifampicin dose resulted in more than a two-fold increase in rifampicin exposure, attributable to its saturable clearance [42]. However, despite the higher rifampicin exposure observed during PK4, there was no significant impact on atazanavir exposure compared to PK3. Recent studies have shown that high dose rifampicin significantly reduces time to sputum conversion, enabling shorter and more effective treatment regimens [43–46]. Our results suggest that increasing rifampicin dose beyond 10 mg/kg would not further affect atazanavir or dolutegravir exposure further. Of note, Kengo et al [47] studied dolutegravir with rifampicin at 35 mg/kg and, whereas they found no additional impact on its clearance compared to rifampicin 10 mg/kg, they reported lower bioavailability, although this did not warrant any dosing adjustment. It is unclear if the lack of effect in our study is due to the lower rifampicin dose or the protective effect of atazanavir/ritonavir. DERIVE has several limitations. First, participants were already stable and suppressed on atazanavir at baseline. However, individuals who fail first-line antiretroviral therapy, necessitating switch to alternative ART may have a period of increased susceptibility to opportunistic infections like tuberculosis. Therefore, assessing the safety of rifampicin and dose-escalated ATV/r in individuals who have recently switched regimen is important. Safety aspects related to tuberculosis could not be evaluated in this study. It is noteworthy that DERIVE participants had an average weight approximately 15 kg higher than observed in tuberculosis patients. Additionally, tuberculosis is treated with combinations of antituberculosis drugs, including isoniazid, which is an inhibitor of CYP3A4 [48]. Therefore, the pharmacokinetics and hepatotoxicity profile may differ when ATV/r is co-administered with the full antituberculosis regimen. Furthermore, we measured liver enzymes for the 7-week study period, but the full TB treatment regimen necessitates 6 months of rifampicin. We may have underestimated the potential long-term effects of the drug combination. Another limitation was that the PK4 visit occurred a week after the rifampicin dose escalation, which might have been insufficient to reach full induction. In total, 88% of DERIVE participants were female. However, existing literature reported higher incidence of AEs in females [49] and elevated CYP3A4 activity [50] relative to males; hence it is reassuring that the suggested dose was well tolerated and above target in our population. Finally, interpretation drew attention to the need for data-driven clinical targets to determine therapeutic concentrations.

## CONCLUSION

DERIVE demonstrates that the induction effect of rifampicin can be overcome by doubling the dose of ATV/r to 300/100 twice daily. This increase in dose was well tolerated, with only transient and asymptomatic grade 1 adverse events observed in a few participants. These results suggest that dose escalation of ATV/r may be a viable strategy for managing drug interactions in patients receiving rifampicin-based therapy.

## Supplementary Data

Supplementary materials are available at *Clinical Infectious Diseases* online. Consisting of data provided by the authors to benefit the reader, the posted materials are not copyedited and are the sole responsibility of the authors, so questions or comments should be addressed to the corresponding author.

## Supplementary Material

ciad700_Supplementary_Data

## Notes

***Acknowledgments.*** The study team is very grateful to the study participants. They appreciate the contributions of the following individuals: M. Boffito, C. van Halsema, A. Szubert, E. Wilkins, R. Kamoga, R. Kabasingo, M. Namasinga, P. Hellen Masiga, J. Namusanje, J. Kobusingye, D. Odoch, P. Ssebunya, C. Nasaazi. K. Gausi acknowledges her postdoctoral fellowship funders Harry Crossley Postdoc Fellowship.

***Financial support*.** This project is part of the EDCTP2 program supported by the European Union (grant number RIA2016MC-1606-VirTUAL).
