# Supplementary material for: Pharmacokinetics and Safety of Twice-daily Ritonavir-boosted Atazanavir With Rifampicin
Source: Clin Infect Dis. 2023 Nov 20;78(5):1246–55. doi: 10.1093/cid/ciad700 (PMC11093668; doi:10.1093/cid/ciad700)
Supplement: ciad700_Supplementary_Data [file ciad700_supplementary_data.docx]

## Supplementary File

## Protease inhibitor assay method

Atazanavir, dolutegravir, and ritonavir were analysed in human plasma with a validated multiplex liquid chromatography tandem mass spectrometry assay, which consisted of a liquid-liquid extraction followed by high performance liquid chromatography with tandem mass spectrometry detection. Atazanavir-d5, dolutegravir-d4 and ritonavir-d6 were used as internal standards. The extraction procedure was followed by liquid chromatographic separation using a Poroshell 120 EC-C18, 50 x 3 mm, 2.7µm analytical column. An isocratic mobile phase containing Mobile Phase A (mixture of water, methanol, and formic acid; 90:10:0.1, v/v) and Mobile Phase B (acetonitrile with 0.1% formic acid) at a ratio of 1 to 1 was used at a flowrate of 400 µL per minute with a 1 to 3 split at the source. The total run time was 4 minutes and the retention times for atazanavir, dolutegravir, ritonavir and their respective internal standards were 1.2, 1.0, and 2.8 minutes, respectively. An AB Sciex API 4000 mass spectrometer at unit resolution in the multiple reaction monitoring mode was used to monitor the transition of the protonated precursor ions. 705.4, 710.4, 420.1, 424.1, 721.4, and 727.4 to the product ions 335.2, 168.1, 277.2, 279.1, 140.1, and 302.2 for atazanavir, atazanavir-d5, dolutegravir, dolutegravir-d4, ritonavir, and ritonavir-d6, respectively. The calibration curves fitted quadratic regressions (weighted by 1/x concentration) over the range 0.030 to 10.0 mg/L for atazanavir and dolutegravir, and of 0.005 to 2.50 mg/L for ritonavir. The accuracy (%Nom) and percentage coefficient of variation (precision) statistics of the low, medium, and high-quality controls were between 99.7 and 108.7%, and below 8.8% during validation.

## Ritonavir pharmacokinetics results

The pharmacokinetics parameters of dolutegravir are presented in **Table 5**, and the median plasma concentration-time profile in **Figure 5**. In brief, the exposure of dolutegravir was higher with the dose escalation of ATV/r C_tau,_ and AUC_24_ were 2- and 1.6-folds higher compared to the standard dose (PK2 vs. PK3). The doubling of the rifampicin dose did not appear to affect dolutegravir exposure.

Table S1: Ritonavir pharmacokinetic parameters

| PK parameter | Geometric mean (CI 90%) | | | | Geometric Mean Ratio (CI 90%) | | | | |
| --- | --- | --- | --- | --- | --- | --- | --- | --- | --- |
|  | **ATV/r 300/100 QD (PK1)** | **ATV/r 300/100 QD + RIF 600 (PK2)** | **ATV/r 300/100 BID + RIF 600 (PK3)** | **ATV/r 300/100 BID + RIF 1200 (PK4)** | **PK2** vs **PK1** | **PK3** vs **PK1** | **PK4** vs **PK1** | **PK4** vs **PK3** | |
| C_tau_ (mg/L) | 0.068  (0.060- 0.077) | 0.0038  (0.0032- 0.0045) | 0.038  (0.029- 0.049) | 0.031  (0.023- 0.042) | **0.056**  **(0.048 - 0.065)** | 0.56  (0.46- 0.68) | 0.46  (0.35-0.60) | 0.83  (0.67- 1.0) |  |
| AUC_24_ (mg·h/L) | 9.5  (8.4 - 11) | 1.3  (1.0 - 1.6) | 7.5  (6.0- 8.5) | 5.6  (4.4 - 7.2) | **0.13**  **(0.11 - 0.17)** | 0.75  (0.65- 0.87) | 0.591  (0.49- 0.71) | 0.784  (0.65- 0.95) |  |
| C_max_ (mg/L) | 1.2  (1.04 - 1.35) | 0.34  (0.28 - 0.41) | 0.79  (0.67 - 0.93) | 0.63  (0.48 - 0.81) | **0.28**  **(0.23 - 0.35)** | **0.67**  **(0.56- 0.79)** | **0.53**  **(0.43- 0.65)** | **0.80**  **(0.63- 1.0)** |  |
| Clearance/F (L/h) | 11  (9.34 - 11.9 | 80  (64 - 99) | 28  (23 - 34) | 35.6  (28- 46) | **7.6**  **(6.18 -9.21)** | **2.65**  **(2.3- 3.1)** | **3.4**  **(2.8- 4.1)** | **1.3**  **(1.1- 1.5)** |  |
| T_1/2_ (h) | 5.7  (5.4- 6.1) | 2.3  (2.2- 2.5) | 2.2  (2.1- 2.3) | 2.3  (2.2- 2.5) | **0.41**  **(0.38- 0.44)** | **0.38**  **(0.37- 0.40)** | **0.41**  **(0.39- 0.43)** | **1.1**  **(1.0- 1.1)** |  |


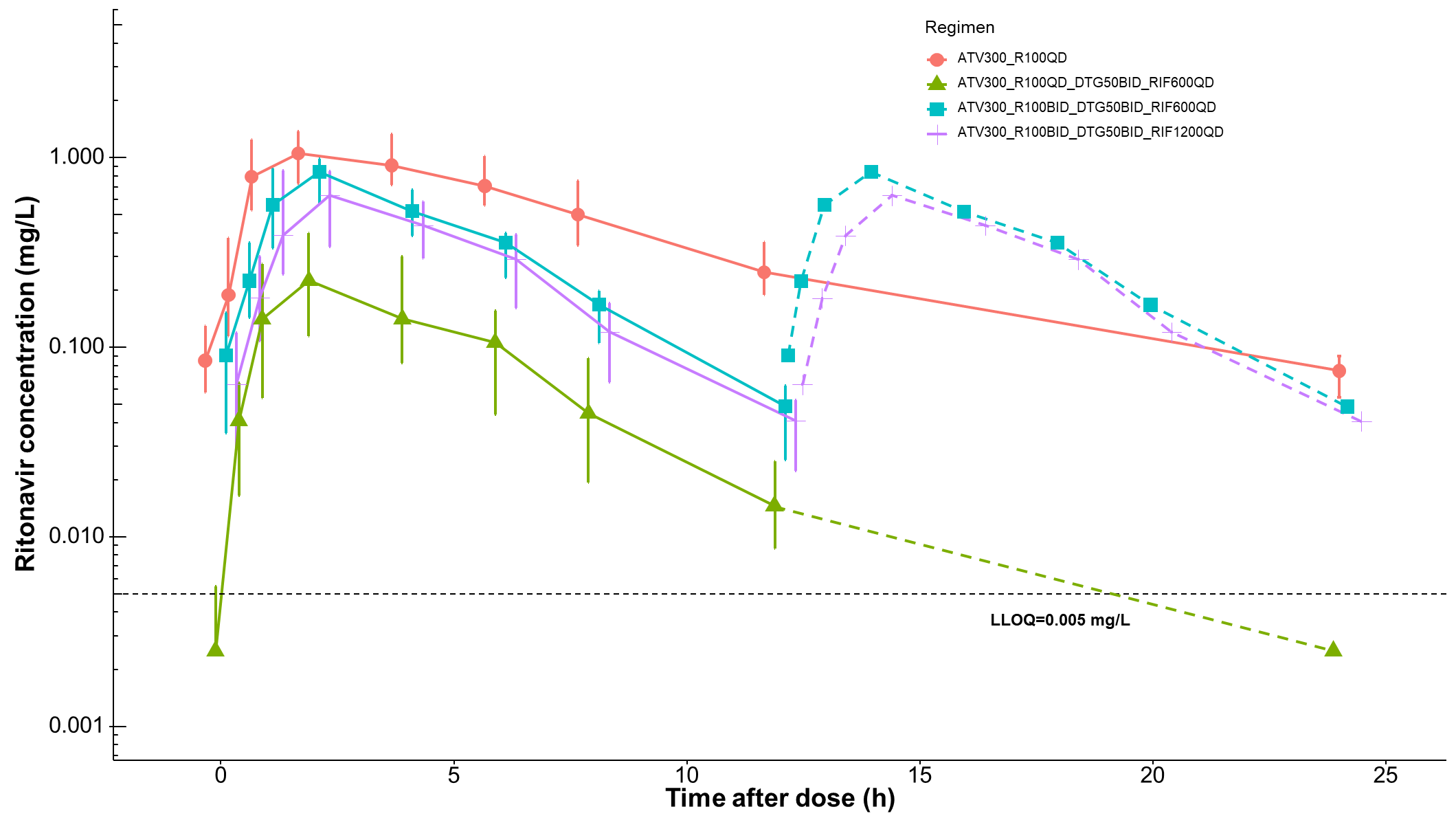


*Figure S1: Ritonavir concentration-time profile (median and interquartile range) across the 4 PK visits*The solid lines present observed data while the dashed lines present a repetition *of the first 12 hour*s *for twice-daily dosing and the* extrapolated *24-hour concentration for the once*-*daily dosing profile* to ease the visual interpretation of the results*. For twice*-*daily dosing the predose* (0 h) and the 12-hours concentration are *not exact*ly the same, *hence the dashed line does not overlap with the solid one. The black horizontal dashed lines represent ritonavir lower limit of quantification of the assay.*
